# Supplementary material for: Sequence of antihypertensive medications used in preterm infants with hypertension: A cross-sectional study
Source: PLoS One. 2025 Apr 22;20(4):e0320002. doi: 10.1371/journal.pone.0320002 (PMC12013942; doi:10.1371/journal.pone.0320002)
Supplement: S1 Table — Represents 120 unique patients. Patients can be included multiple times in the calculation of these summary statistics. (DOCX) [file pone.0320002.s001.docx]

**Supplemental Table 1. Demographics and Baseline Clinical Characteristics of all encounters, by first-line therapy received.** Represents 120 unique patients. Patients can be included multiple times in the calculation of these summary statistics.

| **Characteristic** | **Propranolol**, N = 82*^1^* | **Captopril**, N = 19*^1^* | **Esmolol**, N = 12*^1^* | **Enalapril**, N = 8*^1^* | **Clonidine**, N = 7*^1^* | **Hydralazine**, N = 3*^1^* | **Sodium nitroprusside**, N = 3*^1^* |
| --- | --- | --- | --- | --- | --- | --- | --- |
| Gender |  |  |  |  |  |  |  |
| Female | 29 (36%) | 9 (47%) | 9 (75%) | 1 (12%) | 1 (14%) | 0 (0%) | 1 (33%) |
| Male | 52 (64%) | 10 (53%) | 3 (25%) | 7 (88%) | 6 (86%) | 3 (100%) | 2 (67%) |
| Unknown | 1 | 0 | 0 | 0 | 0 | 0 | 0 |
| Race |  |  |  |  |  |  |  |
| Asian | 1 (1.3%) | 0 (0%) | 0 (0%) | 0 (0%) | 0 (0%) | 0 (0%) | 0 (0%) |
| Black or African American | 35 (45%) | 7 (39%) | 5 (45%) | 1 (12%) | 5 (71%) | 1 (33%) | 0 (0%) |
| Hispanic or Latino | 0 (0%) | 0 (0%) | 0 (0%) | 1 (12%) | 0 (0%) | 0 (0%) | 0 (0%) |
| Other | 2 (2.6%) | 0 (0%) | 0 (0%) | 0 (0%) | 0 (0%) | 0 (0%) | 0 (0%) |
| White | 39 (51%) | 11 (61%) | 6 (55%) | 6 (75%) | 2 (29%) | 2 (67%) | 3 (100%) |
| Unknown | 5 | 1 | 1 | 0 | 0 | 0 | 0 |
| Ethnicity |  |  |  |  |  |  |  |
| Hispanic/Latino | 1 (1.3%) | 2 (13%) | 0 (0%) | 1 (17%) | 0 (0%) | 0 (0%) | 0 (0%) |
| Non-Hispanic/Latino | 75 (99%) | 13 (87%) | 11 (100%) | 5 (83%) | 7 (100%) | 3 (100%) | 3 (100%) |
| Unknown | 6 | 4 | 1 | 2 | 0 | 0 | 0 |
| Age at discharge (days) | 40 (22, 79) | 103 (87, 134) | 24 (14, 45) | 90 (69, 158) | 247 (225, 302) | 40 (28, 148) | 30 (18, 40) |
| Gestational age at birth (weeks) | 33.0 (29.4, 34.0) | 27.0 (26.1, 33.6) | 35.7 (33.2, 36.3) | 35.0 (35.0, 35.5) | 25.0 (24.8, 26.6) | 24.6 (24.4, 25.3) | 33.6 (32.8, 34.8) |
| Unknown | 15 | 9 | 0 | 3 | 0 | 0 | 0 |
| Birthweight (g) | 1,750 (1,043, 2,355) | 760 (558, 1,320) | 2,740 (2,354, 3,200) | NA (NA, NA) | 677 (493, 815) | 804 (804, 804) | 2,195 (2,195, 2,195) |
| Unknown | 39 | 12 | 7 | 8 | 0 | 2 | 2 |
| Percent of SBPs above the 95th percentile 1 day prior to treatment (%; Dionne, Abitbol, and Flynn [2012]) | 7 (2, 17) | 7 (0, 37) | 6 (1, 22) | 1 (0, 7) | 0 (0, 0) | 38 (19, 57) | 12 (12, 13) |
| Unknown | 35 | 12 | 4 | 4 | 6 | 1 | 1 |
| Percent of SBPs above the 90th percentile 1 day prior to treatment (%; Task Force on Blood Pressure Control [1987]) | 27 (15, 39) | 0 (0, 2) | 19 (15, 28) | 3 (1, 7) | 13 (4, 25) | NA (NA, NA) | 95 (95, 95) |
| Unknown | 61 | 14 | 9 | 4 | 0 | 3 | 2 |
| *^1^* n (%); Median (IQR) | | | | | | | |
