# Supplementary material for: Sequence of antihypertensive medications used in preterm infants with hypertension: A cross-sectional study
Source: PLoS One. 2025 Apr 22;20(4):e0320002. doi: 10.1371/journal.pone.0320002 (PMC12013942; doi:10.1371/journal.pone.0320002)
Supplement: S2 Table — (DOCX) [file pone.0320002.s002.docx]

**Supplemental Table 2. Summary statistics for those included in the primary analysis (n = 134) versus those included in the sensitivity analysis of patients with a discharge diagnosis code of primary critical congenital heart disease (n = 21).**

| **Characteristic** | **Primary analysis** | **Primary critical congenital heart disease** |
| --- | --- | --- |
|  | **N = 134***^1^* | **N = 21***^1^* |
| Gender |  |  |
| Female | 50 (38%) | 4 (19%) |
| Male | 83 (62%) | 17 (81%) |
| Unknown | 1 |  |
| Race |  |  |
| Asian | 1 (0.8%) | 0 (0%) |
| Black or African American | 54 (43%) | 9 (43%) |
| Hispanic or Latino | 1 (0.8%) | 0 (0%) |
| Other | 2 (1.6%) | 0 (0%) |
| White | 69 (54%) | 12 (57%) |
| Unknown | 7 |  |
| Ethnicity |  |  |
| Hispanic/Latino | 4 (3.3%) | 0 (0%) |
| Non-Hispanic/Latino | 117 (97%) | 21 (100%) |
| Unknown | 13 |  |
| Age at discharge (days) | 54 (23, 114) | 64 (28, 82) |
| Gestational age at birth (weeks) | 32.5 (28.4, 34.5) | 33.10 (32.00, 34.25) |
| Unknown | 27 | 5 |
| Birthweight (g) | 1,614 (890, 2,321) | 1,260 (1,020, 1,860) |
| Unknown | 70 | 12 |
| Percent of SBPs above the 95th percentile 1 day prior to treatment (%; Dionne, Abitbol, and Flynn [2012]) | 7 (1, 21) | 5 (0, 8) |
| Unknown | 63 | 8 |
| Percent of SBPs above the 90th percentile 1 day prior to treatment (%; Task Force on Blood Pressure Control [1987]) | 18 (4, 37) | 9 (0, 42) |
| Unknown | 93 | 13 |
| *^1^* n (%); Median (IQR) | | |
