# Supplementary material for: Sequence of antihypertensive medications used in preterm infants with hypertension: A cross-sectional study
Source: PLoS One. 2025 Apr 22;20(4):e0320002. doi: 10.1371/journal.pone.0320002 (PMC12013942; doi:10.1371/journal.pone.0320002)
Supplement: Attachment — Submitted filename: Response to Reviewers.docx [file pone.0320002.s005.docx]

**Editorial comments**

*1. Please ensure that your manuscript meets PLOS ONE's style requirements, including those for file naming.*

Headings and subheadings within the abstract and main text have been re-formatted to comply with PLOS ONE’s style requirements. In-text citations have been re-formatted to comply with PLOS ONE’s style requirements. Table 1 has been re-located to the paragraph after it is first referenced. Author affiliations have been updated to match PLOS ONE’s style requirements (see more details below).

*2. Please amend your list of authors on the manuscript to ensure that each author is linked to an affiliation. Author’s affiliations should reflect the institution where the work was done (if authors moved subsequently, you can also list the new affiliation stating “current affiliation:…” as necessary).*

Author affiliations have been updated to match examples displayed in the PLOS ONE style template. Current affiliations for Alejandro D. Perez and Mary-Carty Pittman have been added (denoted by #a and #b, respectively). Author credentials have been removed.

3. *Please include a caption for table 2.*

We thank the reviewer for this comment. However, we were slightly confused as to which caption the reviewer was referring to because we did not have a Table 2 in the main manuscript. We did realize that we neglected to include the Figure captions in the manuscript, and we have updated the manuscript to include the Figure captions after their first mention in the text, in line with PLOS guidelines.

*4. We note that you have indicated that there are restrictions to data sharing for this study. For studies involving human research participant data or other sensitive data, we encourage authors to share de-identified or anonymized data. However, when data cannot be publicly shared for ethical reasons, we allow authors to make their data sets available upon request. For information on unacceptable data access restrictions, please see* [*http://journals.plos.org/plosone/s/data-availability#loc-unacceptable-data-access-restrictions*](http://journals.plos.org/plosone/s/data-availability#loc-unacceptable-data-access-restrictions)*.*

*If there are ethical or legal restrictions on sharing a de-identified data set, please explain them in detail (e.g., data contain potentially identifying or sensitive patient information, data are owned by a third-party organization, etc.) and who has imposed them (e.g., a Research Ethics Committee or Institutional Review Board, etc.). Please also provide contact information for a data access committee, ethics committee, or other institutional body to which data requests may be sent.*

*If there are no restrictions, please upload the minimal anonymized data set necessary to replicate your study findings to a stable, public repository and provide us with the relevant URLs, DOIs, or accession numbers. Please see http://www.bmj.com/content/340/bmj.c181.long for guidelines on how to de-identify and prepare clinical data for publication. For a list of recommended repositories, please see https://journals.plos.org/plosone/s/recommended-repositories. You also have the option of uploading the data as Supporting Information files, but we would recommend depositing data directly to a data repository if possible.*

Even though our de-identified dataset has been classified as Not Human Subjects Research, we are prevented from widely sharing the dataset due to restrictions placed by the data owners. We have updated the last paragraph in our “Materials and Methods” section with instructions for requesting the data. We reproduce that text below for the reviewer’s convenience:

“This study was determined to be Not Human Subjects Research by Auburn University and the University of Alabama at Birmingham. The data from these electronic medical records was delivered from the University of Alabama at Birmingham to Auburn University on January 4th, 2023. Study investigators never had access to information that could identify participants, either during or after data collection. The study authors are prevented from sharing the data publicly due to restrictions placed upon use of the data by the data owners. To initiate a request for the dataset, please contact [ResearchData@uabmc.edu](mailto:ResearchData@uabmc.edu) and reference request “i2b2.lds.inpatient infants receiving antihypertensives w/dx codes <37 pt set”. The corresponding author ([matthew_loop@auburn.edu](mailto:matthew_loop@auburn.edu)) will do everything he can to facilitate obtainment of the data for reasonable requests.”

*5. Please remove your figures from within your manuscript file, leaving only the individual TIFF/EPS image files, uploaded separately.*

The figures have been removed from the manuscript file.

*6. Please include captions for your Supporting Information files at the end of your manuscript, and update any in-text citations to match accordingly. Please see our Supporting Information guidelines for more information:* [*http://journals.plos.org/plosone/s/supporting-information*](http://journals.plos.org/plosone/s/supporting-information)*.*

We have placed the captions for all supplemental tables at the end of the manuscript file.

*7. Please review your reference list to ensure that it is complete and correct. If you have cited papers that have been retracted, please include the rationale for doing so in the manuscript text, or remove these references and replace them with relevant current references. Any changes to the reference list should be mentioned in the rebuttal letter that accompanies your revised manuscript. If you need to cite a retracted article, indicate the article’s retracted status in the References list and also include a citation and full reference for the retraction notice.*

The reference list has been reviewed for completeness/correctness. We have removed one reference from 2000 at the recommendation from one of the reviewers, in favor of a newer review paper from 2024. The new review paper is

Gaffar S, Ramanathan R, Easterlin MC. Common clinical scenarios of systemic hypertension in the NICU. Neoreviews. 2024 Jan 1;25(1):e36–49.

**Reviewer 1**

*1. The research article is novel and well written.*

We thank the reviewer for their kind words.

*2. However,there are a few corrections to be made. Introduction:pharmacologic management should consistently be used instead of pharmacotherapy.*

We thank the reviewer for this suggestion, and we have made this language change throughout the manuscript. We have replaced every use of “therapy” with “pharmacologic management” or “medication,” when “medication” seemed to be the more appropriate term.

*3. Regarding data availability, kindly elaborate more on the kind of restrictions that apply.*

Please see response to editorial comment #4 above.

*4. Discussion;first paragraph, the author should add citations for the mechanism of action of propranolol, it's bioavailability and side effects.*

We thank the reviewer for noting this oversight. We have hadded citations for these claims in the first paragraph of the Discussion.

*5. The author should add the date on reference 12 especially when it was accessed.*

We thank the reviewer for their comment. We have decided to update the citation from simply the website to a manuscript that describes the implementation of i2b2. We believe this change will be more helpful and instructive to the reader than our original citation. We have reproduced the citation below for the reviewer’s convenience.

Murphy SN, Weber G, Mendis M, Gainer V, Chueh HC, Churchill S, et al. Serving the enterprise and beyond with informatics for integrating biology and the bedside (i2b2). J Am Med Inform Assoc. 2010 Mar;17(2):124–30.

*6. Ethics:the approval letter to conduct this study should be attached.*

We have uploaded the letter documenting a conversation with the head of the Auburn University IRB confirming that our dataset did not constitute Human Subjects Research. This document should be available in the resubmission as well.

**Reviewer 2**

*1. Perez et al. have performed a cross-sectional study on the sequence of antihypertensive medications used in preterm infants with hypertension. By assessing 751 individuals, they found that propranolol was the most commonly used medication for first-line anti-hypertensive therapy among preterm infants. The manuscript is well-written and the findings are interesting.*

We thank the reviewer for their kind words about our manuscript.

*These are my comments:*
*- The results section could be organized by adding subheadings.*

We thank the reviewer for this suggestion to improve the readability of the Results section. We have added the following subheadings: “Description of the study sample,” “Summary of antihypertensive pharmacologic management,” and “Sensitivity analysis.” We think these subheadings will help guide the reader through the manuscript, and we thank the reviewer again for the suggestion.

*2. A paragraph summarizing the clinical take-home message of this manuscript should be added to the discussion.*

We thank the reviewer for this suggestion. We have added a new pagraph at the end of the Discussion section, and we reproduce it below for the reviewer’s convenience:

“This study’s clinical takeaway is that more systematic focus on tracking how preterm infants with hypertension are treated will lead to improved outcomes for these patients. The large difference in the most common treatment for hypertension between our single-site study (propranolol) and a nationwide study of patients diagnosed with hypertension (hydralazine)[11] highlights how variable treatment practices can be among different health systems. Different treatment practices among health systems are especially large for preterm infants [19]. Further complicating the picture is the fact that one quarter of infants who are treated for hypertension never receive a formal diagnosis, which makes the process of accurately summarizing pharmacologic management strategies difficult. Variation among health systems, and ultimately patient outcomes, can be reduced by: (i) systematically documenting the existing pharmacologic management; (ii) identifying the optimal management strategy; and (iii) producing guidelines and recommendations to disseminate evidence on optimal pharmacologic management. Since there are limited data on management strategies for preterm infants, clinicians in each health system are tasked with using their best clinical judgement. Our study extends prior research on overall exposure to antihypertensive medications and documents the sequencing of medications for pharmacological management, which is step (i) above. Future rigorous studies to build systematic evidence for the pharmacologic management of hypertension in preterm infants will lead to increased standardization of care and, ultimately, better outcomes for these patients.”

*3. The references prior to 2010 could be updated with those after 2010 since they provide more up-to-date findings.*

We thank the reviewer for their comment. We have replaced the review article from 2000 with an updated review article from 2024. The new review article is:

Gaffar S, Ramanathan R, Easterlin MC. Common clinical scenarios of systemic hypertension in the NICU. Neoreviews. 2024 Jan 1;25(1):e36–49.
